# Interventions for adolescent alcohol consumption in Africa: protocol for a scoping review including an overview of reviews

**DOI:** 10.1186/s13643-021-01642-4

**Published:** 2021-03-29

**Authors:** Alice M. Biggane, Eleanor Briegal, Angela Obasi

**Affiliations:** 1 Department of International Public Health, Liverpool School of Tropical Medicine, Pembroke Place, Liverpool, UK; 2 AXESS Sexual Health, Liverpool University Hospitals NHS Foundation Trust, Liverpool, UK

**Keywords:** Scoping review, Alcohol use interventions, Africa, Adolescents

## Abstract

**Background:**

Harmful alcohol use is a leading risk to the health of populations worldwide. Within Africa, where most consumers are adolescents, alcohol use represents a key public health challenge. Interventions to prevent or substantially delay alcohol uptake and decrease alcohol consumption in adolescence could significantly decrease morbidity and mortality, through both immediate effects and future improved adult outcomes. In Africa, these interventions are urgently needed; however, key data necessary to develop them are lacking as most evidence to date relates to high-income countries. The purpose of this review is to examine and map the range of interventions in use and create an evidence base for future research in this area.

**Methods:**

In the first instance, we will conduct a review of systematic reviews relevant to global adolescent alcohol interventions. We will search the Cochrane Database of Systematic Reviews, MEDLINE (Ovid), CINAHL, Web of Science, Global Health and PubMed using a broad search. In the second instance we will conduct a scoping review by drawing on the methodological framework proposed by Arksey and O’Malley. We will search for all study designs and grey literature using the Cochrane Database of Systematic Reviews, MEDLINE (Ovid), CINAHL, Web of Science and Global Health, Google searches and searches in websites of relevant professional bodies and charities. An iterative approach to charting, collating, summarising and reporting the data will be taken, with the development of charting forms and the final presentation of results led by the extracted data. In both instances, the inclusion and exclusion criteria have been pre-defined, and two reviewers will independently screen abstracts and full text to determine eligibility of articles.

**Discussion:**

It is anticipated that our findings will map intervention strategies aiming to reduce adolescent alcohol consumption in Africa. These findings are likely to be useful in informing future research, policy and public health strategies. Findings will be disseminated widely through peer-reviewed publication and in various media, for example, conferences, congresses or symposia.

**Protocol Registration:**

This protocol was submitted to the Open Science Framework on May 03, 2021. www.osf.io/qnvba

**Supplementary Information:**

The online version contains supplementary material available at 10.1186/s13643-021-01642-4.

## Background

Harmful alcohol use is a leading risk to the health of populations worldwide; it is a significant barrier to achieving many health-related targets of the Sustainable Development Goals (SDGs), including those for maternal and child health, infectious diseases, noncommunicable diseases, mental health and injuries and poisonings [1]. Alcohol use represents a key public health challenge in Africa where it accounts for more deaths and disability-adjusted life years (DALYs) lost than in any other region [1, 2] and twice as many preventable deaths as tobacco [3].

Most alcohol consumers in Africa are adolescents and young people; the use is highly gendered, and adolescent males are at particular risk [4]. Evidence suggests that early alcohol initiation (aged < 14 years) predicts alcoholism in middle age [1] and is potentially a more powerful precursor to alcoholism than excess drinking in early adulthood [1]. Adolescents are more vulnerable to alcohol-related harm per volume than adults [1], and those who drink are more likely than their elders to engage in heavy episodic drinking (HED) (> 60 g alcohol at least once in the preceding month), which the WHO (World Health Organization) has identified as the most deleterious drinking pattern [5]. Alcohol use in adolescents is associated with alterations in verbal learning, visual–spatial processing, memory and attention as well as with deficits in development and integrity of the grey and white matter of the central nervous system [6]. These neurocognitive alterations are associated with behavioural, emotional, social and academic problems in later life [7, 8]. Further, alcohol consumption in adolescence is associated with sexual risk taking [9], adverse HIV outcomes, self-harm, suicide and the perpetration of sexual violence [4].

Interventions to prevent or substantially delay alcohol uptake and decrease alcohol consumption in adolescence could significantly decrease morbidity and mortality, through both immediate effects and future improved adult outcomes [4, 10]. These interventions are urgently needed in Africa; however, key data necessary to develop them are lacking as most evidence to date relates to high-income countries (HICs) [10–13]. We are aware of only one systematic review which included an evaluation of interventions to reduce adolescent alcohol consumption [14]; it featured one study from Africa [15]. This evidence gap was highlighted by Das et al. in their 2016 global overview of systematic reviews regarding adolescent substance abuse interventions including alcohol, in which they cautioned “there is a dire need for rigorous, higher quality evidence especially from low- and middle-income countries” [16]. This call has since been echoed by others [17]. The current review complements this work and specifically aims to map and characterise the specific adolescent alcohol interventions which have been used in Africa.

### Types of intervention

Adolescent alcohol use is shaped by a complex range of factors acting at multiple levels in the environments in which adolescents grow and develop [18]. These levels of influence are commonly categorized in socio-ecological frameworks [18, 19] as macro-system (e.g. policies, societal beliefs and cultures), community level (neighbourhood risks and resources), micro-system (households, schools, peer networks), and individual level (gender, age, socioeconomic status).

**Fig. 1:**
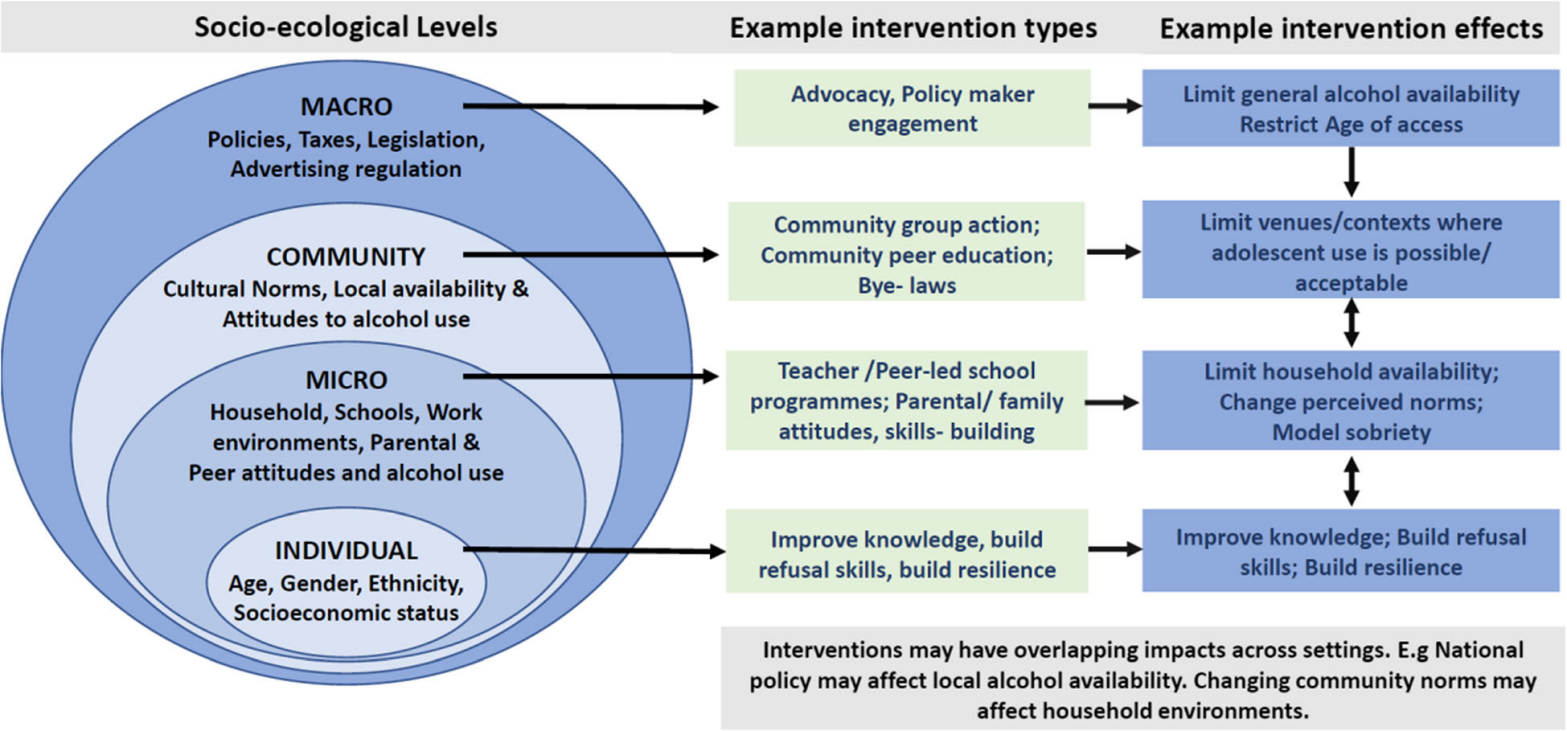
Intervention levels and example mechanisms of action

Figure 1 illustrates how interventions seek to exert effects or modify factors at one or more of these levels and how strategies used to deliver the interventions within settings (e.g. teachers and/or peers as educators in school-based programmes) vary. Theoretical models underpinning proposed mechanisms of action for intervention (e.g. Stages of Change model vs. Theory of Planned Behaviour) also vary. We will assess the available evidence for each type of intervention and identify evidence gaps to inform future research and implementation.

## Methods

This review protocol has been registered within the Open Science Framework database (registration number: www.osf.io/qnvba). Further, this review protocol is also being reported in accordance with the reporting guidance provided in the Preferred Reporting Items for Systematic Review and Meta-Analysis Protocols (PRISMA-P) 2015 statement as appropriate which can be found in Additional File 1 [20, 21].

The review will be conducted in two stages. First, in stage one, the proposed overview of systematic reviews will capture systematic reviews published since 2000 to complement Das et al.’s 2016 overview of systematic reviews [16] and provide the most up to date syntheses of the evidence base. This overview of reviews will be reported in accordance with the Preferred Reporting Items for Systematic Review and Meta-Analysis (PRISMA) 2020 [20, 21].

Second, in stage two, the proposed scoping review of peer reviewed and grey literature published since 2000 will identify interventions and gaps in the evidence base relating to adolescent alcohol interventions in Africa. This will be reported in accordance with the PRISMA Extension for Scoping Reviews checklist (PRISMA-ScR) [22].

The methodologies for each of the above two stages are described in what follows. In both stages, interventions will be categorized by setting, delivery model and theoretical construct. Adolescents are defined as those aged 10–19 years; however, since many studies target youth (aged 15–24 years), we will include reviews and interventions targeting older groups if adolescents are also included. If possible, we will stratify our findings by age. Otherwise, we will report the combined results for adolescents and youth as representative of the population of interest. If we identify a new, relevant systematic review providing good quality evidence for appropriate interventions in Africa, we will at that point discuss the need for the scoping review and proceed as deemed appropriate.

### Stage 1: Overview of systematic reviews

We will identify and review recent Cochrane and non-Cochrane systematic reviews of randomised or non-randomised controlled trials, which fully or partly addressed alcohol interventions for adolescents. For the purpose of this review, we have defined a systematic review as a review of evidence based on a clearly formulated question, to identify and critically appraise relevant research by following a systematic, explicit and repeatable methodology [23].

### Eligibility criteria

We will develop a comprehensive search strategy to review the available literature underpinned by our pre-defined inclusion criteria (Table 1).

**Table 1:** Overview of systematic review inclusion criteria

Inclusion criteria	
Published since January2000	
Any language publication	
Intervention to prevent, delay or otherwise modify alcohol use among adolescents	
Population includes 10–24-year olds	

Our pre-defined exclusion criteria are as follows:

- No information on an alcohol use intervention
- Duplicate publications
- Reviews other than systematic, e.g. narrative, scoping
- Grey literature
- Published before 2000
- Interventions that were not purposely developed to target adolescent alcohol consumption
- Interventions that were exclusively targeting individuals aged 25 years or more

### Identifying relevant studies

We will search the Cochrane Database of Systematic Reviews, MEDLINE (Ovid), CINAHL, Web of Science, Global Health and PubMed for publications published from January 2000 onwards, using a broad search strategy building on that outlined by Das et al. [16] in their 2016 overview. This will include a combination of appropriate keywords, medical subject headings (MeSH terms) and free text terms; an outline of our search strategy for PubMed is available in Additional File 2; it will be updated accordingly for the other databases. We will also examine cross-references and bibliographies of included publications to identify additional sources of information. If required, we will contact the publication’s lead author to clarify or seek additional information. All articles identified from the literature search will be screened by two reviewers independently. First, titles and abstracts of articles returned from the initial searches will be screened based on the eligibility criteria outlined above. Second, full texts will be examined in detail and screened for eligibility. Third, references of all considered articles will be hand-searched to identify any relevant publication missed in the search strategy. Any disagreements on selection of reviews will be resolved via discussion and if needed the input of a third reviewer. A flow chart showing studies included and excluded at each stage of the screening process will be included in the full publication [24].

### Extracting and charting the data

After retrieval of the full texts of all the reviews that meet the inclusion criteria (Table 1), data from each review will be extracted, independently by two reviewers, in a standardised form using Microsoft Excel. Data we will collect includes but is not limited to:

- Author(s), year of publication, publication type, study location
- Study populations—characteristics and locations
- Aims of study
- Intervention details building on the TIDieR Format [25] (name, rationale/theory, materials, provider, mode, context (e.g. school/community/clinic), intensity and duration, tailoring, modification, fidelity)
- Comparator (if any)
- Target demographics (gender, age, i.e. older/younger adolescents (10–14/15–19))
- Geographical location—country
- Setting (e.g. urban/rural)
- Outcome measured
- Measurement of treatment effects
- Inclusion and exclusion criteria
- Risk of bias tool

### Types of intervention

As shown in Fig. 1, the types of intervention will vary, and we anticipate that some may be complex interventions operating at more than one ecological level. For example, community-based interventions aimed at decreasing alcohol availability for adolescents may be combined with school-based programmes targeting individual knowledge. The latter maybe delivered by teachers or peer educators.

These elements and any other relevant information regarding the intervention programmes (socio-ecological level, setting, delivery mechanism, target group, behaviour change theory), acceptability and costs will be extracted. When there is missing data, we will attempt to contact the original authors to obtain the relevant information. We do not have any pre-planned data assumption or simplifications. We will extract pooled effect size for the outcomes reported by the review authors with 95% confidence intervals (CIs). We will assess and report, in duplicate, the quality of included reviews using the 11-point assessment of the methodological quality of systematic reviews (AMSTAR-2) criteria [26]. We will report the final results using the Preferred Reporting Items for Systematic Reviews and Meta-Analyses (PRISMA) reporting tool [24].

### Data analysis

We will analyse the data arising from all included publications to create an overview of the various adolescent alcohol interventions being used global and their reported effectiveness and location. We plan to analyse the data using descriptive statistics via Microsoft Excel and report the findings narratively, using tables to characterise key features, interventions and findings. We will also seek to identify whether interventions were exclusively designed to target alcohol consumption or were part of a wider substance abuse or healthcare intervention. Where possible, we will explore both the variations and overlap that may exist in findings of the reviews, as well as issues such as the numbers of studies included, date ranges covered by the reviews, sample sizes, target populations and settings. However, we will be adaptive to the data we extract and the subsequent analysis as appropriate.

### Scoping review

A scoping review will allow us to identify and map the range and type of interventions as described in Fig. 1 [24, 27]. A strength of this type of review is that, in addition to published articles, we will also search for grey literature, such as reports and guidance documents as it is possible that some of the information being sought (i.e. descriptions of alcohol interventions in use) for our target population are documented in non-traditional forms of scientific publications. In designing our scoping review protocol, we draw on Arksey and O’Malley’s methodological framework [27] and its amendments [28, 29] as follows.

#### Identifying the research question

Based on gaps in the literature and the study team’s knowledge of the field these are as follows:

1. What interventions have been used to delay, reduce or otherwise modify alcohol consumption among adolescents in Africa?
2. What are the settings, delivery methods, theoretical bases and reported effectiveness of these interventions?

These questions will be refined, or new ones added, as the researcher team becomes familiar with the literature [27].

#### Identifying relevant studies

We will develop a comprehensive search strategy to review the available literature using the ‘Population–Concept–Context (PCC)’ framework for scoping reviews [30], underpinned by our pre-defined inclusion criteria (Table 2).

**Table 2:** Population Concept Context (PCC) framework providing an overview of the components and characteristics of the research question

Population Concept Context (PPC) Framework—inclusion criteria	
Components	Characteristics
**P**opulation	Adolescent boys and girls (10–19 years of age) in Africa
**C**oncept	Literature with specific focus and/or statements describing alcohol use interventions targeting adolescents and youth in Africa.
**C**ontext	All study designs, reports, blogs, book chapters, editorials and commentaries from the public health field since 2000. There will be no language restrictions

Our pre-defined exclusion criteria are as follows:

- No information on an alcohol use intervention
- Duplicate publications
- Protocol only
- Published before 2000
- Not used in Africa
- Interventions that were exclusively targeting individuals aged 25 years or more

Drawing on the three-step process recommended by JBI [29], we will systematically search the following databases: Cochrane Database of Systematic Reviews, MEDLINE (Ovid), CINAHL, Web of Science and Global Health for relevant publications from the year 2000 onwards. We will also perform targeted searches for grey literature published from the year 2000 onwards, by searching (1) Google, (2) relevant discipline-based listservs (e.g. academic institutes) and (3) the websites of agencies that fund or implement public health interventions in Africa (e.g. ministries of health, charity organisations). Relevant blogs, newsletters, reports and surveys will also be considered.

The draft literature search for MEDLINE (Ovid) can be found in supplementary information Additional File 3, which uses a combination of keywords, MeSH and free text terms; it will be updated accordingly for the other databases. Intervention types will not be included in the search to avoid limiting the results. We will review potentially relevant text words in the titles and abstracts of important papers in the field, thus compiling a list of terms that can be used to inform our search strategy. The literature search will be supplemented by handsearching of the reference lists of included studies for keywords and contacting methodological experts in each field. The search strategy and its iterations will be peer reviewed by a health librarian specialist using the Peer Review of Electronic Search Strategies (PRESS) checklist [31]. There will be no language restrictions and relevant articles will be translated into English as needed.

#### Study selection

All identified records (titles and abstracts) will be collated in a reference manager for de-duplication. The abstracts (and the full sources where abstracts are not available) will be screened by two reviewers to identify relevant literature based on our a priori inclusion criteria. Neither of the review authors will be blind to the journal titles or to the study authors or institutions, after which we will retrieve the full text of all potentially eligible articles, which will also be independently screened. Any disagreements during screening will be resolved via discussion and if needed the input of a third reviewer. The final unique set of records will be imported into an Excel file to facilitate independent screening and log disagreements between reviewers. We will also record reasons for exclusion at the full-text review stage.

We expect that some of the grey literature might subsequently be published elsewhere in the indexed literature. This will be accounted for by cross-checking authors’ names across grey literature and index literature results to identify potential duplicates.

#### Charting the data

We will develop a charting form to aid the collection and recording of key information using Excel, this will be done in duplicate. We will record the following:

- Author(s), year of publication, publication type, study location
- Study populations
- Aims of study
- Intervention type, and comparator (if any); duration of the intervention
- Demographics (gender, age, i.e. older/younger adolescents (10–14/15–19))
- Geographical location—country
- Setting (e.g. urban/rural, school/community/clinic)
- Methodology
- Outcome measured
- Important results

The information from research-based and non-research-based publications will be collected in separate extraction forms. Additional categories that may emerge during data extraction will be added accordingly.

#### Collating, summarising and reporting the results

We will combine all relevant findings from the data retrieved across the various sources to create a useful summary which identifies and maps relevant interventions and their characteristics. This will include general and specific descriptions of the interventions, the population targeted, the delivery methods, the reported effectiveness and lessons learned where possible. Further, we will also extract relevant data surrounding development of the intervention and resources required. We plan to analyse the data using descriptive statistics via Microsoft Excel and report the findings narratively. If appropriate, we will include tables describing key features. If possible, and dependent on the number of studies retrieved and included, we will include a geographical map showing areas in which interventions have been used. We will also look for overlap and variations between the studies in terms of intervention type, results, setting, population targeted and follow-up timeframe. However, we will be adaptive to the data we extract and the subsequent analysis as appropriate. It should be noted that this study will not assess the quality of evidence and therefore cannot comment on the generalisability and robustness of individual studies [27]. We will report the final results using the PRISMA Extension for Scoping Reviews checklist (PRISMA-ScR) [22].

### Amendments

Any amendments to this protocol when conducting the study will be outlined in Open Science Framework and reported in the final manuscript.

## Discussion

Our scoping review including an overview of reviews will systematically identify and map the interventions used to target adolescent alcohol use in Africa. Both stages of our review will be of value to a range of stakeholders in the field of adolescent alcohol use. Our characterisation of the different interventions that exist, the degree to which each has been implemented and tested and the gaps and priority research questions identified will be relevant to a variety of audiences including researchers, public health practitioners, policy makers and charity organisations.

Publication of this research protocol is in keeping with good, transparent research practise, as it reduces the risk of bias and selective reporting while providing an opportunity to strengthen our proposed review.

We do not anticipate any practical or operational issues arising that will affect the performance of this study as our research team has experience and knowledge of both the subject matter and the methodology. We will make our data available to other researchers by request. One potential limitation of this study is the difficulties that exist in categorising adolescents in terms of age; however, by including studies with participants up to the age of 24 years and stratifying our results as possible, we should capture all relevant populations as previously outlined in this protocol.

As there are no human participants involved, there will be no requirement for ethical approval. Patients and/or the public were not involved in the design of this protocol; however, the authors will work with patients and members of the public through stakeholder and other PPI research forums in disseminating the findings of the review both in the UK and the Global South.

Findings will be disseminated widely through peer-reviewed publication and in various media, for example, conferences, congresses or symposia. This review will inform other researchers in the field of adolescent health as a standalone piece of work but will also provide a baseline resource which can be used to inform future research planning.

## Supplementary Information

**Additional file 1.**

**Additional file 2.**

**Additional file 3.**

## Data Availability

Not applicable

## Abbreviations

AMSTAR: Assessment of the Methodological Quality of Systematic Reviews
DALYs: Disability-adjusted life years
HED: Heavy episodic drinking
HIC: High-income countries
JBI: Joanna Briggs Institute
PCC: Population, Concept, Context
PRESS: Peer Review of Electronic Search Strategies
PRISMA: Preferred Reporting Items for Systematic Reviews and Meta-Analysis
PRISMA-P: Preferred Reporting Items for Systematic Review and Meta-Analysis Protocols
PRISMA-ScR: Preferred Reporting Items for Systematic Reviews and Meta-Analyses Extension for Scoping Reviews
SDGs: Sustainable Development Goals
WHO: World Health Organization
