# Supplementary material for: Interventions for adolescent alcohol consumption in Africa: protocol for a scoping review including an overview of reviews
Source: Syst Rev. 2021 Mar 29;10:88. doi: 10.1186/s13643-021-01642-4 (PMC8006360; doi:10.1186/s13643-021-01642-4)
Supplement: Supplementary file 2 — Additional file 2. [file 13643_2021_1642_MOESM2_ESM.docx]

## Additional File 2. Overview of reviews- Search Strategy

The following is a combination of MeSH terms and free text for a search in PubMed. We will filter by systematic review published since January 2000. We will edit this search strategy as appropriate for the other databases.

In June 2020 this search produced 426 articles on PubMed. Given the small number of articles retrieved, we will filter for interventions in the screening process.

| **Concept** | **Search Terms** |
| --- | --- |
| Population | 1. Child [MeSH terms]  2. Adolescent [MeSH terms]  3. Young Adult [MeSH terms]  4. school-aged children [ free-text term]  5. OR 1-4 |
| Alcohol | 6. Alcoholic Beverages [MeSH terms]  7. Alcohol Drinking [MeSH terms]  8. Alcohol-Related Disorders [MeSH terms]  9. Alcohol-Induced Disorders [MeSH terms]  10. Alcoholism [MeSH terms]  11. Underage Drinking [MeSH terms]  12. Ethanol [MeSH terms]  13. Binge Drinking [MeSH terms]  14. Alcoholic Intoxication [MeSH terms]  15. alcohol* [free-text term]  16. intoxicat* [ free-text term]  17. drunk [free-text term]  18. OR 6-17 |
| Final Search Strategy | 20. 5 and 18 |
