# Supplementary material for: Interventions for adolescent alcohol consumption in Africa: protocol for a scoping review including an overview of reviews
Source: Syst Rev. 2021 Mar 29;10:88. doi: 10.1186/s13643-021-01642-4 (PMC8006360; doi:10.1186/s13643-021-01642-4)
Supplement: Supplementary file 3 — Additional file 3. [file 13643_2021_1642_MOESM3_ESM.docx]

## Additional File 3. Scoping review- Search Strategy

The following is a combination of MeSH terms and free text for a search in Medline. We will search for relevant literature published since January 2000. We will edit this search strategy as appropriate for the other databases and in accordance with results from the overview of reviews if necessary.

In June 2020 this search produced 3,650 articles on Medline. To avoid limiting the search results during, we will filter for interventions in the screening process.

| **Concept** | **Search Terms** |
| --- | --- |
| Population | 1. Child [MH terms]  2. Adolescent [MH terms]  3. Young Adult [MH terms]  4. school-aged children* [ free-text term]  5. OR 1-4 |
| Alcohol | 6. Alcoholic Beverages [MeSH terms]  7. Alcohol Drinking [MeSH terms]  8. Alcohol-Related Disorders [MeSH terms]  9. Alcohol-Induced Disorders [MeSH terms]  10. Alcoholism [MeSH terms]  11. Underage Drinking [MeSH terms]  12. Ethanol [MeSH terms]  13. Binge Drinking [MeSH terms]  14. Alcoholic Intoxication [MeSH terms]  15. alcohol* [free-text term]  16. intoxicat* [ free-text term]  17. drunk [free-text term]  18. OR 6-17 |
| Africa | 20. Africa+ [MH terms]  21. Africa* [free-text term]  22. 20 OR 21 |
| Final Draft Search Strategy | 5 AND 19 AND 22 |
